# Detecting and characterizing high-frequency oscillations in epilepsy: a case study of big data analysis

**DOI:** 10.1098/rsos.160741

**Published:** 2017-01-18

**Authors:** Liang Huang, Xuan Ni, William L. Ditto, Mark Spano, Paul R. Carney, Ying-Cheng Lai

**Affiliations:** 1 School of Physical Science and Technology, Lanzhou University, Lanzhou, Gansu 730000, People's Republic of China; 2 School of Electrical, Computer and Energy Engineering, Arizona State University, Tempe, AZ 85287, USA; 3 School of Biological and Health Systems Engineering, Arizona State University, Tempe, AZ 85287, USA; 4 Department of Physics, Arizona State University, Tempe, AZ 85287, USA; 5 College of Sciences, North Carolina State University, Raleigh, NC 27695, USA; 6 Pediatric Neurology and Epilepsy, Department of Neurology, University of North Carolina, 170 Manning Drive, Chapel Hill, NC 27599-7025, USA

**Keywords:** nonlinear dynamics, electroencephalogram, epileptic seizures, empirical modedecomposition, big data analysis, high-frequency oscillations

## Abstract

We develop a framework to uncover and analyse dynamical anomalies from massive, nonlinear and non-stationary time series data. The framework consists of three steps: preprocessing of massive datasets to eliminate erroneous data segments, application of the empirical mode decomposition and Hilbert transform paradigm to obtain the fundamental components embedded in the time series at distinct time scales, and statistical/scaling analysis of the components. As a case study, we apply our framework to detecting and characterizing high-frequency oscillations (HFOs) from a big database of rat electroencephalogram recordings. We find a striking phenomenon: HFOs exhibit on–off intermittency that can be quantified by algebraic scaling laws. Our framework can be generalized to big data-related problems in other fields such as large-scale sensor data and seismic data analysis.

## 1. Introduction

Big data analysis [1–6], a frontier field in science and engineering, has broad applications ranging from biomedicine and smart health [7, 8] to social behaviour quantification and energy optimization in civil infrastructures. For example, in biomedicine, vast electroencephalogram (EEG) or electrocorticogram (ECoG) data are available for the analysis, detection and possibly prediction of epileptic seizures (e.g. [9–16]). In a modern infrastructure viewed as a complex dynamical system, large-scale sensor networks can be deployed to measure a number of physical signals to monitor the behaviours of the system in continuous time [17–19]. In a modern city, smart cameras are placed in every main street to monitor the traffic flow at all times. In a community, data collected from a large number of users carrying various mobile and networked devices can be used for community activity prediction [20]. In wireless communication, big datasets are ubiquitous [21, 22]. In all these cases, monitoring, sensing or measurements typically result in big datasets, and it is of considerable interest to detect behaviours that deviate from the norm or the expected.

In this paper, we develop a general and systematic framework to detect hidden and anomalous dynamical events, or simply anomalies, from big datasets. The mathematical foundation of our framework is Hilbert transform and instantaneous frequency analysis. The reason for this choice lies in the fact that complex dynamical systems are typically nonlinear and non-stationary. For such systems, the traditional Fourier analysis is limited because, fundamentally, they are designed for linear and stationary systems. Windowed Fourier analysis may be feasible to generate patterns in the two-dimensional frequency–time plane pertinent to characteristic events, but two-dimensional feature identification is difficult. By contrast, the features generated by the empirical mode decomposition (EMD) methodology are one dimensional, which are easier to be identified computationally. The Hilbert transform and instantaneous frequency-based analysis have proved to be especially suited for data from complex, nonlinear and non-stationary dynamical systems [23–25]. The challenge is to develop a mathematically justified and computationally reasonable framework to uncover and characterize ‘unusual’ dynamical indicators that may potentially be precursors to a large-scale, catastrophic dynamical event of the system.

The general principle underlying the development of our big data-based detection framework is as follows. First, we develop an efficient procedure for preprocessing big datasets to exclude erroneous data segments and statistical outliers. Next, we exploit a method based on a separation of time scales, the EMD method [23, 24], to detect anomalous dynamical features of the system. Owing to its built-in ability to obtain from a complex, seemingly random time series a number of dominant components with distinct time scales, the method is anticipated to be especially effective for anomaly detection. We pay particular attention to the challenges associated with big datasets. Finally, we perform statistical analysis to identify and characterize the anomalies and articulate their implications.

As a concrete example to illustrate the general principle of our big data analysis framework, we address the detection of high-frequency oscillations (HFOs), which are local oscillatory field potentials of frequencies greater than 100 Hz and usually have a duration less than 1 s [26–37]. Oscillations between 100 and 200 Hz are called ripples and occur most frequently during episodes of awake immobility and slow-wave sleep. The HFOs in this range are believed to play an important role in information processing and consolidation of memory [38, 39]. Pathologic HFOs (with frequency larger than 200 Hz, or fast ripples [40]) reflect fields of hyper-synchronized action potentials within small discrete neuronal clusters responsible for seizure generation. They can be recorded in association with interictal spikes only in areas capable of generating recurrent spontaneous seizures [41]. Thus detecting fast ripples can be useful in locating the seizure onset zone in the epileptic network [29, 42, 43], and this was verified previously using datasets from a wide variety of patients [32]. In particular, it was found that almost all fast-ripple HFOs were recorded in seizure-generating structures of patients suffering from medial or polar temporal-lobe epilepsy, indicating that the ripples are a specific, intrinsic property of seizure-generating networks in these brain areas. The pathologic HFOs and their spatial extent can potentially be used as biomarkers of the seizure onset zone, facilitating decisions as to whether surgical treatment would be necessary. Besides their role in locating the seizure onset zone, HFOs may also reflect the primary neuronal disturbances responsible for epilepsy and provide insights into the fundamental mechanisms of epileptogenesis and epileptogenicity [44, 45].

Traditional methods such as the Fourier transform and spectral analysis assume stationarity and/or approximate the physical phenomena with linear models. These approximations may lead to spurious components in their time–frequency distribution diagrams if the underlying signal is non-stationary and nonlinear. EMD is a technique [23] to specifically deal with non-stationary and nonlinear signals. Given such a signal, EMD decomposes it into a small number of modes, the intrinsic mode functions (IMFs), each having a distinct time or frequency scale and preserving the amplitude of the oscillations in the frequency range. The decomposed modes are orthogonal to each other, and the sum of all modes gives the original data. The ease and accuracy with which the EMD method processes non-stationary and nonlinear signals have led to its widespread use in various applications such as seismic data analysis [23], chaotic time series analysis [24, 46], neural signal processing in biomedical sciences [47], meteorological data analysis [48] and image analysis [49]. We develop an EMD-based method to detect HFOs. Owing to its built-in ability to pick out from a complex, seemingly random time series a number of dominant components of distinct time scales, the method is especially effective for the detection of HFOs. We finally perform a statistical analysis and find a striking phenomenon: HFOs occur in an on–off intermittent manner with algebraic scaling. In addition to HFOs, our framework can detect population spikes, oscillations in the frequency range from 10 to 50 Hz, as well as distinct and independent IMFs.

As pathologic HFOs reveal dynamical coherence within small discrete neuronal clusters responsible for seizure generation, a good understanding and accurate detection of HFOs may bring the grand goal of early seizure prediction one step closer to reality and would also improve the localization of the seizure onset zone to facilitate decision-making with regard to surgical treatment. Not only does our method illustrate, in a detailed and concrete way, an effective way to analyse big datasets, our finding also has potential impact in biomedicine and human health.

There were existing works on applying the EMD/Hilbert transform method to neural systems. Earlier the method was applied to analysing biological signals and performing curve fitting [50], and a combination of EMD, Hilbert transform and smoothed nonlinear energy operator was proposed to detect spikes hidden in human EEG data [51]. Subsequently, it was demonstrated [52] that the methodology can be used to analyse neuronal oscillations in the hippocampus of epileptic rats *in vivo* with the result that the oscillations are characteristically different during the pre-ictal, seizure onset and ictal periods of the epileptic EEG in different frequency bands. In another work [53], the EMD/Hilbert transform method was applied to detecting synchrony episodes in both time and frequency domains. The method was demonstrated to be useful for decomposing neuronal population oscillations to gain insights into epileptic seizures [54], and EMD was used for extracting single-trial cortical beta oscillatory activities in EEG signals [55]. The outputs of EMD, i.e. the IMFs, were demonstrated to be useful for EEG signal classification [56]. Our work differs from these previous works in that we address the issue of detecting HFOs and uncovering the underlying scaling law.

## 2. Results

### 2.1. Pretreatment of datasets

High-sampling (12 KHz), multichannel (32–64 channels), continuous recordings of local field potentials in freely moving rodents present unique technical challenges. Although most channels continue to record over a four- to six-week period, over time the integrity of the signal degrades and electrode recording may come off- and online. To this end, it is important to preprocess data files to exclude gaps in data. This in itself is challenging due to the large size of each dataset (approx. 5 TB), variability during recordings of local field potentials, and gaps in data. Here, we develop a fully automated statistical method. The resulting ‘data-mining’ algorithm is general and we expect it to be useful for dealing with other massive datasets.

For our study, we examine EEG data taken from a rat model of the approach to epilepsy. The typical size of a binary file in our database is about 600–700 MB. Each file belongs to a certain channel (specified by a channel number) and a specific time duration (specified by a file number). We regard the channel and file numbers as two orthogonal dimensions and plot the contour of a suitable statistical quantity (to be discussed below) in the two-dimensional plane, so the data of one rat (approx. 5 TB) can be represented by a single contour plot. The whole process can be programmed to be highly parallelized, providing a global overview of the raw EEG data.

Let $d_{i}$, (i=1,…,L) be the value of the EEG signal for a single sample, where *L* is the number of samples in a binary file. In the experiment, each value $d_{i}$ is recorded as a 16-bit integer, so $d_{i}\in [-N,N-1]$, where $N=2^{15}$ and, typically we have $L∼3\times 10^{8}$ samples. We then examine the values of $d_{i}$ and count the number of each value present in the file, which results in an array $n_{j}$, j=−N,…,N. Repeated values over some periods in the oscillation pattern lead to corrupted files, which can be due to recording errors—this, indeed, happened in our experiment. In general, when hours or even minutes of bad recordings of zeros are encountered, the number $n_{0}$ of zeros in the file counted will increase rapidly. These features of abnormal recordings will be utilized to exclude the corrupted files.

**Figure 1.**
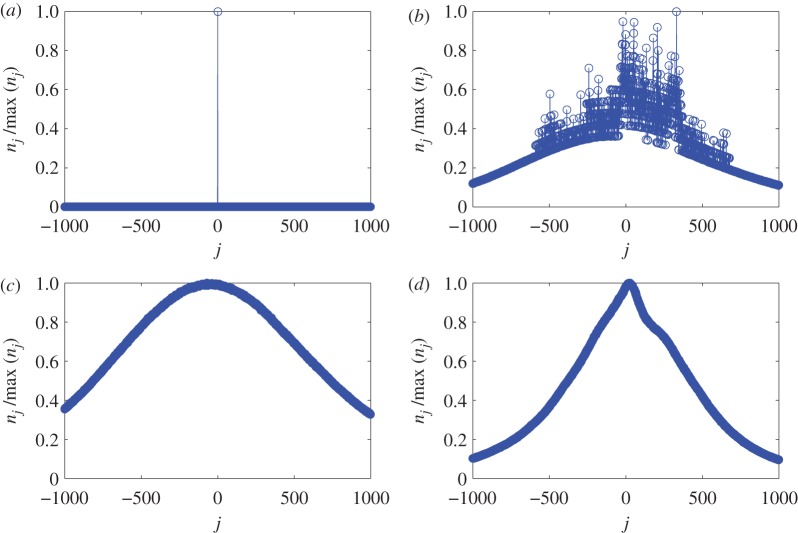
Pretreatment of massive data from rat EEG. Different types of distributions of $n_{j}$ for Rat004 channel 02. For each panel, the *y*-axis is normalized by the maximum of $n_{j}$. The four panels correspond to: (*a*) a corrupted file with a large number of zeros (file no. 20), (*b*) a bad recording with repetitions of oscillating patterns (file no. 73), (*c*) a normal file without transitions (file no. 77) and (*d*) a file containing a seizure (file no. 99).

Figure 1 shows four typical types of $n_{j}$ distributions obtained from channel 02 of Rat004 composed of 229 data files. For binary files that have large numbers of continuous zeros, for example, file number 20, the distribution is shown in figure 1*a*. An example of a corrupted file is where a specific pattern of oscillations is embedded repeatedly in most of the data in the file. The corresponding distribution is shown in figure 1*b*. The distributions from normal data files qualified for dynamical analysis are shown in figure 1*c*,*d*. The distribution in figure 1*c* is approximately Gaussian. However, seizure events can cause distortions from the Gaussian distribution, as evidenced by figure 1*d* for file number 99 where a clinically certified seizure is present. We observe that the distribution becomes somewhat narrowed (as compared with the case of no seizure) and slightly asymmetrical.

After obtaining the distribution for each file, we define and compute a statistical quantity for each file, and assemble the files within the same channel according to this quantity, as follows. Let $s_{k}=n_{k-N}-n_{k-N-1}$ (k=1,…,2N), where $s_{k}$ represents the difference between two neighbouring counts, and let $\sigma_{s}^{2}=1/(2N-1)\sum_{k=1}^{2N}{\left(s_{k}-\bar{s}\right)}^{2}$ be the variance, where $\bar{s}$ is the mean value of $s_{k}$. Note that $n_{j}$ is not normalized and their sum is the data length *L* of the file. Denoting ${\left\langle n\right\rangle }_{j}$ as the smoothed curve of $n_{j}$, we have ${\left\langle n\right\rangle }_{j}∼L$. The fluctuations, as characterized by $s_{k}$, are in general proportional to ${\left\langle n\right\rangle }_{j}$. As a result, the variance $\sigma_{s}^{2}$ is positively correlated with *L*, e.g. a larger value of *L* would result in a larger value of $\sigma_{s}^{2}$.

**Figure 2.**
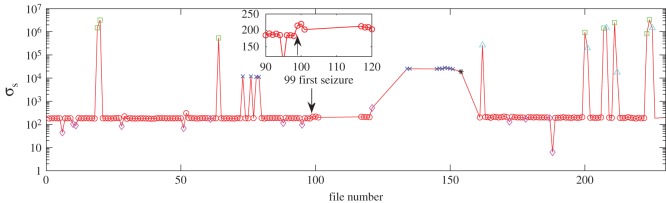
Statistical properties of massive data from rat EEG. Standard deviation $\sigma_{s}$ for Rat004 channel 02. Red circles denote the normal files; green squares are the files with large numbers of zeros; blue crosses are corrupted files; pink diamonds are small files; cyan triangles are small files with many zeros; black star is small corrupted files. The arrow marks the file 99 which has the first seizure. The inset shows the enlarged area around file 99 on a linear scale.

Thus for small files that are normal in all other aspects, we will have smaller $\sigma_{s}$ values, which can be clearly identified from figure 2 as the points below the majority. This figure also shows some points with extremely large $\sigma_{s}$ values. These points correspond to the binary files with large numbers of continuous zeros (figure 1*a*). The corrupted data all have $\sigma_{s}$ values in the range $10^{4}∼10^{5}$ (figure 1*b*) which can be excluded readily, as shown in figure 2. A transition in $\sigma_{s}$ at file number 99 (when the first seizure occurred) is observed.

**Figure 3.**
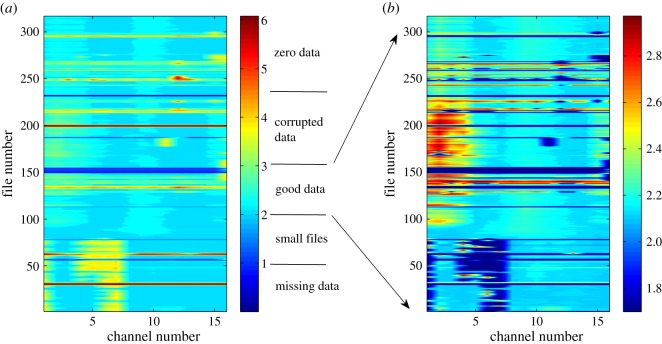
Contour representation of massive data from rat EEG. Contour plot of $\log_{10}\sigma_{s}$ for Rat001. (*a*) The whole $\sigma_{s}$ range. Different types of data are classified according to the value of $\log_{10}\sigma_{s}$. In (*b*), the contour is for values of $10^{2}\leq \sigma_{s}\leq 10^{3}$ (good data), and the remaining values of $\sigma_{s}$ are set to 50 so that the dark blue area marks all abnormal data.

By applying the same procedure to multiple channels, the massive EEG data from one rat can be expressed using a single contour plot of $\sigma_{s}$, as shown in figure 3. We can immediately identify different types of data in terms of the values of $\log_{10}\sigma_{s}$. It should be noted that for different ‘abnormal’ situations, e.g. small files, contamination with zeros or corrupted data, there can be different methods of remedy based on examining different aspects particular to the data. However, our method is general and efficient in that a single indicator is effective at distinguishing the different types of abnormalities in the files during the preprocessing stage of the massive database for further dynamical and statistical analysis.

### 2.2. Empirical mode decomposition analysis of electroencephalogram data

We conducted extensive tests of applying the EMD procedure to EEG data (see Material and methods). A general finding is that the resulting IMFs in different frequency ranges possess statistical features that are relevant to certain brain activities, demonstrating that the EMD methodology can be effective for probing the dynamical origins of epileptogenesis. For example, typically the frequencies of the first 5 IMFs are about 5 kHz, 2 kHz, 1 kHz, 500 Hz, 200 Hz, 100Hz. As the sampling frequency is 12 kHz, the first three modes correspond to mostly noise contained in the original EEG data. The fourth to sixth modes, whose frequencies lie in the range between 50 Hz and 800 Hz represent the intrinsic dynamical evolution of the underlying brain system.

**Figure 4.**
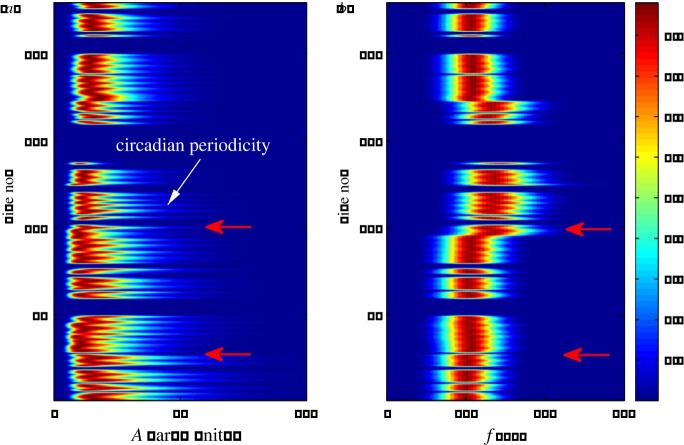
Typical EMD representation of massive rate EEG data. (*a*) Contour plot of normalized distribution of amplitude *A* (in arbitrary units) varying in time of a particular EMD mode of interest (IMF5, approx. 200 Hz) for channel 11 in CA1 of EEG recording of a rat over a two-month period. The all-blue region indicates corrupted files. Each file is a 7 h recording at the sampling frequency 12 kHz. Thus, the vertical axis ‘file no.’ indicates time. The distribution is calculated and then normalized by the maximum value for each file. The rat underwent surgery between file 28 and file 29, and the first seizure occurred in file 99, as indicated by the red arrows. The comb-like structure indicates the circadian periodicity. (*b*) Normalized distribution of the frequency *f* of the mode.

Our procedure for analysing long EEG data thus consists of performing the EMDs to obtain different IMFs, calculating the amplitudes and frequencies of the IMFs that are deemed to reveal the dynamical evolution of the brain, and performing statistical analysis of the on-intervals for the IMFs in a proper frequency range. An example is shown in figure 4, where the distributions of the amplitude and frequency of an IMF for a particular channel of two months' duration are shown. The entire dataset was divided into 230 files, each containing 7 h of EEG recording. Note that when performing the EMD, the data are broken into small segments, e.g. 5 s for each segment, to make the computation more efficient. To calculate the IMFs, a 0.5 s segment is included at each end of the 5 s segment to eliminate the edge effect so that the IMFs can be accurately determined. From figure 4, we can distinguish the changes in the rat brain activity, such as stimulation and the occurrence of the first seizure. Recurrent seizures are not so clearly visible in this plot. Another apparent feature revealed is the circadian periodicity. The EEG recording has a 24 h periodicity because of the circadian activity or of the external treatment of the rat such as feeding, etc., which also changes the frequency and amplitude of each decomposed IMF. As each file is 7 h long, the circadian periodicity indicates a periodicity of three (or four) files in the plot, which is apparent from the comb-like structure in the plot, especially in figure 4*a*, where two adjacent comb teeth have a separation of about three files.

### 2.3. Detection of high-frequency oscillations and population spikes

**Figure 5.**
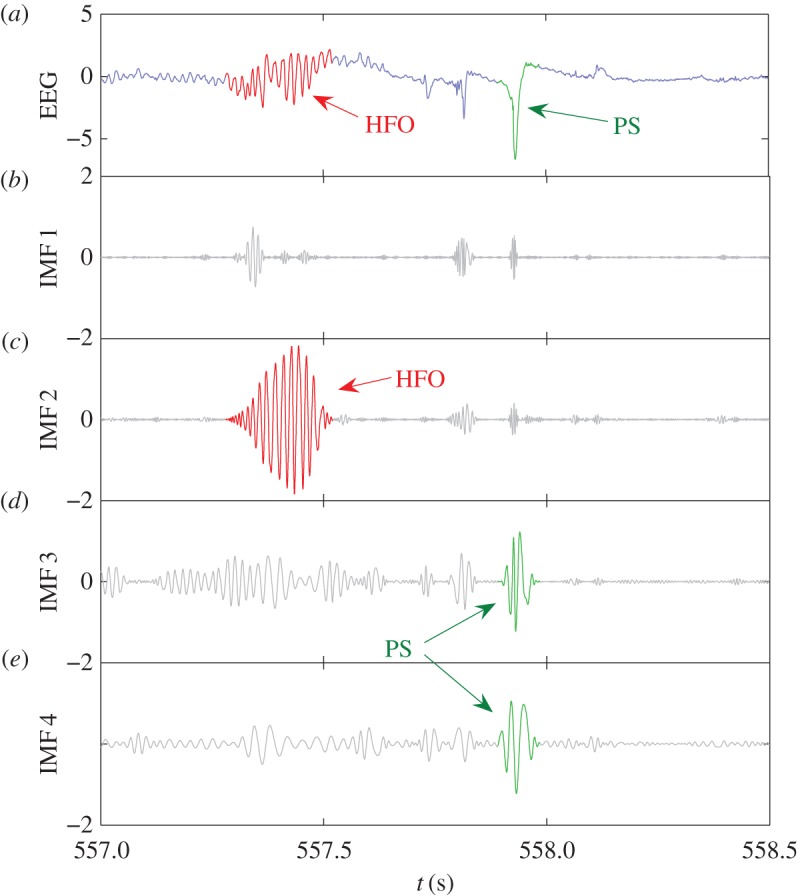
Example of EMD-based HFO detection from EEG data. (*a*) A 1.5 s segment of normalized EEG data containing an HFO and a population spike. (*b*–*e*) The IMFs in the frequency range of interest. The HFO is revealed in IMF 2 and the population spike is revealed in IMF 3 and IMF 4.

To illustrate the procedure of detection of HFOs and population spikes, we reduce the sampling rate so that these dynamical events can be visualized clearly. Note that, when the sampling rate is reduced, the noisy components are effectively filtered out, so the first few IMFs become important. (In the detection and statistics of HFOs and population spikes in the following sections, higher sampling frequency should still be used, in which case the first few IMFs need to be disregarded, as discussed above). Figure 5 shows, for a segment of down-sampled EEG data, the relevant empirical modes. For this dataset, the frequencies are 200–500 Hz for mode 1, 80–200 Hz for mode 2, about 50 Hz for mode 3 and 30 Hz for mode 4. Taking mode 2 as an example, the IMF will have small amplitude if the original EEG data does not contain oscillations in the corresponding frequency range. When the EEG data contains these oscillations, they will be revealed in the corresponding IMF. As HFOs are generally associated with frequencies larger than 80 Hz, they will be revealed in the first two modes. The population spikes (with a time scale of 0.1 s) are decomposed by EMD into oscillations in the frequency range 10–50 Hz, thus they will mainly be manifested in modes 3 and 4. The EEG data in figure 5*a* contains an HFO and a population spike. It is apparent that the HFO and population spike are separated by EMD into different modes and are localized in different time scales, e.g. figure 5*c* for the HFO and figure 5*d*,*e* for the population spike. Thus, the amplitude of the modes evolving in time can be used to detect the HFOs or population spikes, depending on the frequency range of the mode.

Our results thus suggest strongly the feasibility of developing EMD-based algorithms to systematically detect all the HFOs and population spikes. In this regard, we note an existing method of detection of HFOs, which employs short-time energy or line length of the acquired data for HFOs in some small frequency ranges [57]. Our method is capable of detecting HFOs and can be used to distinguish various oscillation profiles. Based on the detected HFOs and population spikes, extensive statistical analyses for the five critical phases during epileptogenesis, namely, pre-stimulation state, pre-seizure state, status epilepticus phase, epilepsy latent period, spontaneous/recurrent seizure period, can be carried out to gain *unprecedented* insights into epileptogenesis.

It can occur that, for a particular signal whose highest frequency component is most significant to the underlying dynamics, the first IMF contains the dominant dynamics with the highest frequency, the next is a lower frequency background to it and so on. However, while the first IMF is the highest frequency component, the corresponding frequency range may not necessarily be relevant to the system dynamics. In fact, we found that typically the first IMF corresponds to noise, and the next IMF contains information about the dynamics of the system. Which IMFs are actually useful and informative depends on the nature of the original signal. More specifically, what EMD does is to decompose the signal into different frequency components through different IMFs, which contain the time varying amplitude and frequency information for each component embedded in the original signal. If the signal is contaminated by noise, the first IMF would be the noise component that contains little information about the underlying dynamics. Our analysis of the massive EEG data indicates that this is indeed the case.

### 2.4. Automated detection and classification of high-frequency oscillations

**Figure 6.**
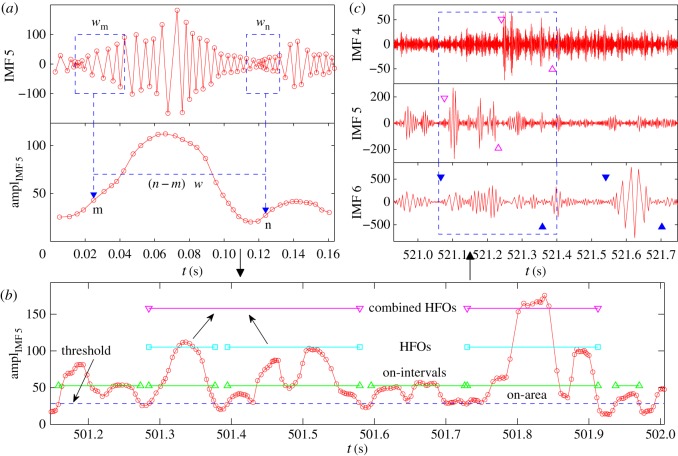
Illustration of HFO detection method. The method consists of three steps. (*a*) Computing the amplitude function from each IMF generated by EMD. The size of the moving window is w=7 periods (indicated by the blue dashed boxes). The time step for the moving window is Δw. (*b*) For each IMF, we locate the on-intervals, find HFOs, and combine adjacent HFOs if they are too close to each other. The blue dashed line is the threshold chosen for the segment of the amplitude function. (*c*) Classifying HFOs in terms of their frequencies, e.g. ripples (solid blue triangles), fast ripples (open magenta triangles) and then combining overlapping HFOs across different IMFs, as shown in the blue dashed box.

Our method to detect, characterize and understand HFOs from EEG recordings consists of the following steps: (i) performing EMD and calculating distinct IMFs, (ii) searching for HFOs based on the amplitudes of IMFs and (iii) classifying HFOs in terms of their frequencies and calculating the statistical properties of HFOs. An illustration of the steps is shown in figure 6.

We first calculate the amplitudes of the IMFs from an automated EMD procedure. We then locate the extrema of one IMF and define the interval between two neighbouring maxima (or minima) to be one period *T*, as shown in figure 6*a*. Unlike the Fourier transform that becomes ineffective in time-series analysis when the signal frequency changes with time, EMD is well suited for generating IMFs whose frequencies vary with time, i.e. when the period is a function of time: T=T(t). We set a moving time window of size *w* and calculate the average IMF amplitude within the window. The window contains a fixed number of IMF periods. As the period varies with time, the actual time span of the window also changes with time. As an example, we show in figure 6*a* two windows $w_{m}$ and $w_{n}$, each containing the same number (7) of IMF periods. Apparently, their sizes are different. The amplitude values are weighted magnitudes and are calculated as $A_{m}=(1/2)\sum_{i\in w_{m}}(x_{i+1}-x_{i})(|y_{i+1}|+|y_{i}|)$, where $x_{i}$ and $y_{i}$ are the position and magnitude of the IMF, respectively, and the factor $\left(x_{i+1}-x_{i}\right)$ is the corresponding weight. The calculation is repeated when the window is moved to the next position by the step size Δw, which is also chosen to contain a certain number of IMF periods. Small values of *w* and Δw result in rapidly oscillating amplitude functions, whereas too large values of *w* and Δw would cause a loss of information. Empirically, the window size can be chosen to include several IMF periods, e.g. six to nine, where the moving forward step size Δw can be set as one.

The next step is to find on-intervals, time intervals when the amplitude values are larger than a certain threshold $A_{c}$, as shown in figure 6*b*. To set a proper threshold, we can pick a segment (e.g. 1 h long) from the amplitude data and calculate the mean *μ* as well as the standard deviation *σ*. One way to set the threshold is $A_{c}=a_{\mu }\mu +a_{\sigma }\sigma$, where $a_{\mu }$ and $a_{\sigma }$ are two adjustable parameters on the order unity. To characterize each on-interval $O_{i}$, we define an on-area $S_{i}$, the area of the IMF in the on-interval above the threshold:

2.1 $$S_{i}=(1/2)\sum_{j\in O_{i}}(x_{j+1}-x_{j})(A_{j+1}+A_{j}-2A_{c}),$$

where $x_{j}$ and $A_{j}$ are the position and magnitude of the amplitude function of the underlying IMF, respectively. The notation $S_{i}$ with the capital *S* should be distinguished from $s_{k}$ that denotes the difference between two neighbouring counts. On-intervals are sorted in the descending order in terms of their on-areas: $S_{1}>S_{2}>\cdots$. The HFOs are identified as those with the largest on-areas, i.e. with longer duration and larger oscillating amplitudes. As the on-area values of HFOs are typically much larger than those of non-HFO on-intervals, the HFOs can be reliably identified as the outliers in the on-area statistics, as follows.

Starting from the most significant on-interval $O_{1}$, we can evaluate its on-area $S_{1}$ with a score function of the remaining on-intervals. If

2.2 $$S_{1}>\alpha E[{\left\{S_{i}\right\}}_{i\geq 2}]+\beta \sqrt{\mathrm{Var}[{\left\{S_{i}\right\}}_{i\geq 2}]},$$

$O_{1}$ will be identified as an HFO, where *α* and *β* are two adjustable parameters, and E[⋅] and Var[⋅] are the expectation and variance functions. This process is repeated until no on-intervals in the remaining sequence satisfy the criterion. Typically, for one IMF, approximately 10% of the on-intervals would be selected as HFOs. Among the remaining HFOs, one can see that some are so close to each other that it is reasonable to combine them. The combinations are carried out wherever the gap *G* between two neighbouring HFOs is small, i.e. when $G<g\cdot \min(T_{{\mathrm{HFO}}_{1}},T_{{\mathrm{HFO}}_{2}})$, where *g* is the parametric gap tolerance ratio and $T_{\mathrm{HFO}}$ is the HFO duration. An example of combining HFOs is shown in figure 6*b*.

**Figure 7.**
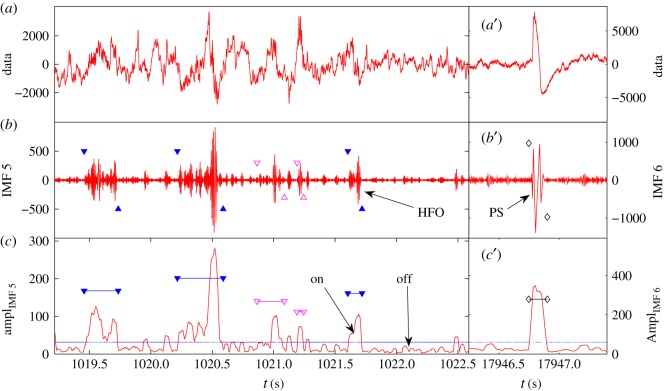
Example of successful HFO and PS detection. (*a*) Original EEG data plot of about 3 s. (*b*) IMF 5 plot with solid blue triangles marking the ripples and open magenta triangles marking the fast ripples. (*c*) The amplitude of IMF 5. The horizontal blue line is the threshold for separating on/off intervals of HFOs. The threshold is calculated from the amplitude data segment of about 1 h. The computational parameters are $a_{\mu }=1$ and $a_{\sigma }=1$. (*a*${\;}^{\prime }$)–(*c*${\;}^{\prime }$) The original data, IMF 6, and its amplitude function, respectively. The black diamonds mark the position of the population spikes.

The HFOs in different frequency ranges are usually responsible for different brain behaviours such as normal information processing and spontaneous seizures. The third step of our procedure is to calculate the frequencies of various HFOs, which is done by locating the starting and ending times of an HFO, finding the number *n* of oscillating periods within it and dividing by its duration: $f=n/T_{\mathrm{HFO}}$. When necessary, we combine overlapping HFOs across different IMFs, as shown in figure 6*c*. When various HFOs have been identified, we can classify them into distinct frequency ranges: low-frequency range (less than 80 Hz), ripples (80–200 Hz, between pairs of solid blue triangles in figure 6*c*) and fast ripples (greater than 200 Hz, between pairs of open magenta triangles in figure 6*c*). HFOs of frequencies lower than 80 Hz are identified as population spikes. An example of identifying and classifying HFOs is shown in figure 7.

### 2.5. Statistical and scaling properties of high-frequency oscillations

As described, the on- and off-intervals associated with HFOs can be determined by setting a threshold value $A_{c}$ in the amplitude function of a given IMF. For a given HFO, an on-area can then be defined, which is the area of the portion of the amplitude function above the threshold. The most significant HFOs are those with the largest on-areas. The on-intervals thus provide a base for characterizing the HFOs. Intuitively, HFOs of various magnitude correspond to ‘islands’ of various sizes above the ‘sea’ level defined by the threshold. To obtain a more complete understanding of HFOs, it is insightful to examine the corresponding ‘undersea’ dynamics (below the threshold). It is computationally feasible to study the dynamical and statistical characteristics of the oscillations below the ‘sea’ level but only within a certain depth.

To illustrate our approach in a concrete way, we take the example in figure 7 and focus on mode 5 because the frequency of this mode lies in a suitable HFO frequency range. From the contour plot of the distribution of amplitude versus file number, figure 4, we see that there are several regions of distinct properties. In particular, the EEG data are relatively stable before the stimulation, say between files 28 and 29. After the stimulation, the data changed characteristically, as can be seen from the amplitude distribution plot in figure 4*a* (indicated by the arrow between files 28 and 29). The first seizure occurred in file 99, and the data are stable for files 29–99. There is a relatively small change between files 51 and 52, as can be seen from the amplitude plot in figure 4*a*, when the rat was actually moved from one cage to another. We have checked other modes and also data from other channels and found a consistency in the specific data segmentation as described. It is thus useful to study these different segments separately: files 1–28 (before stimulation), files 29–51 (between stimulation and first seizure), files 52–94 (pre-ictal phase), files 100–172 (postictal phase but with recurrent seizures in files 105 and 125) and files 175–223 (with recurrent seizures in files 192, 209 and 216). To obtain stable statistics, we temporarily disregard a few files that are in the transitional regime. However, these files may be important in providing possible hints about how the seizure (e.g. the one that occurred around file 99) is developed in terms of the transient dynamics. The specific segmentation scheme of the EEG data is only for one rat, but it is valid for all the channels.

**Figure 8.**
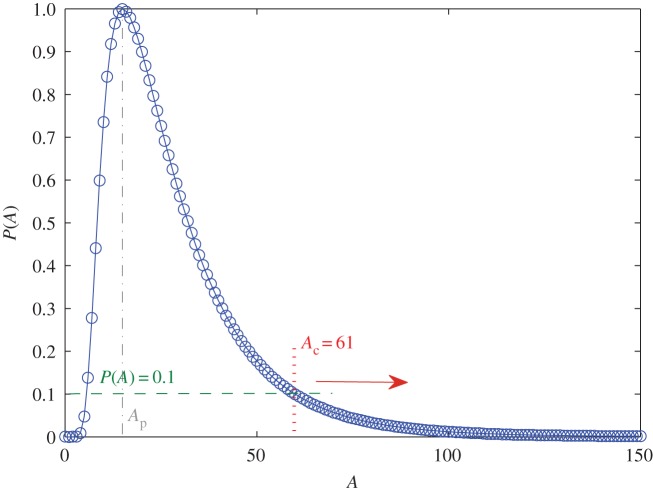
Determination of threshold $A_{c}$. Normalized distribution P(A) of the amplitude *A* for files 1–28 for the same mode as in figure 4, where $A_{p}$ is the value of amplitude at the peak of the distribution. For example, by setting P(A)=0.1 and assuming that $A_{c}>A_{p}$, $A_{c}$ can be determined to be 61. If P(A) takes a smaller value, then $A_{c}$ will be larger.

For different segments the signal amplitudes can have systematic differences, and in certain cases the average amplitude can be, for example, twice larger in one segment than in another. It is thus necessary to determine and set different thresholds in different segments. To do this, first we calculate the normalized distribution of the amplitude *A* in each segment. The distribution for files 1–28 is shown in figure 8. Second, from the peak point defined as $P(A_{p})=1$ (as *P* is normalized to unity), we decrease *P* with small steps, e.g. P=0.98,0.96,…,0.02. For each *P* value, we determine the corresponding threshold $A_{c}$ ($>A_{p}$), as demonstrated in figure 8 for P=0.1, where the corresponding threshold is $A_{c}=61$. Third, for each value of $A_{c}$, we calculate all the on-intervals in this segment from the amplitude function. The distributions of the durations *T* of the on-intervals from different segments are then compared, and various threshold values are set such that the values of $P(A_{c})$ are all identical across the segments.

**Figure 9.**
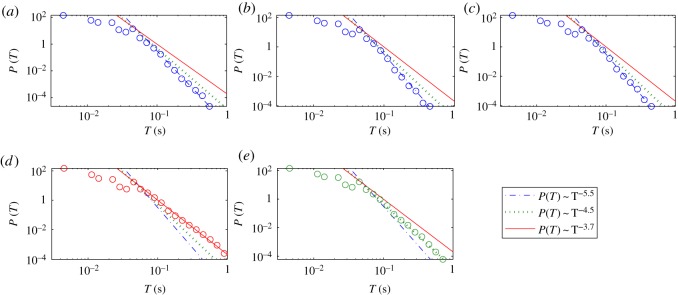
Statistical and scaling behaviours of HFOs. Distributions of on-interval *T* of IMF 5 (channel 11, figure 4): (*a*–*e*) for files 1–28, 29–51, 52–94, 100–172, 175–223, respectively. The numbers of on-intervals are 344 310, 314 698, 431 674, 498 947 and 510 096 for (*a*–*e*), respectively. An algebraic distribution is observed with different exponents for different segments. The exponent for the solid, dotted and dash-dotted lines are −3.7,−4.5 and −5.5, respectively. The threshold $A_{c}$ is chosen such that $P(A_{c})=0.02$ for all the segments.

Figure 9 shows an example of the statistical distribution of the on-intervals for all five segments as specified above. We observe algebraic distributions. For each segment, the algebraic scaling regime extends over at least one order of magnitude in the length of the on-interval. The distributions for the three segments before the first seizure have approximately the same algebraic scaling exponent, as shown in figure 9*a*–*c*, although the amplitude value varies continuously for these segments, as shown in figure 4. After the first seizure, the exponent becomes smaller, indicating more on-intervals with longer durations. For example, in figure 9*d*, for on-intervals with T∼0.4 s, the probability is 100 times larger than those before the seizure. For files 175–223, however, the exponent is somewhat increased, signifying a decrease in the probability of longer on-intervals, but it is still larger than the probability before the seizure. We have systematically checked on-interval statistics for different thresholds. The algebraic scaling and the qualitative difference among the scaling exponents from different segments are robust with respect to variations of the threshold in a certain range (e.g. $P(A_{c})$ ranging from 0.02 to 0.3).

As many on-intervals (especially the long ones) correspond actually to HFOs, our discovery of the algebraic scaling suggests that the HFOs appear more active and sustaining associated with seizure activities, which is consistent with previous observations [29, 31, 32]. In general, an algebraic scaling indicates a hierarchical organization in the underlying dynamics, which in our case, suggests such an organization in the brain neuronal activities. For example, the local synchrony among discrete neuron clusters may vary in hierarchical scales. The fact that approximately the same algebraic scaling exponent occurred before the seizure indicates that, after the stimulation, while evolving toward epilepsy, the underlying dynamics behave the same as in the normal brain. This could be due to the latency effect of the stimulation. The development into epilepsy, however, occurs in a relatively short period, similar to the cascading phenomena associated with earthquakes [58].

We have also checked other channels, which are so selected that they belong to different (neural) correlation clusters. For some channels, behaviours similar to those in figure 9 are observed, but significant deviations occur for some other channels. This may be because the HFO and the seizure onset zone are usually highly localized [32]. As a result, only within a proper range of this zone can the HFOs be detected. As the distance between the neighbouring channels is quite small (approx. 0.25 mm), the HFO and the underlying neuronal activity could be revealed in a small subset of channels.

**Figure 10.**
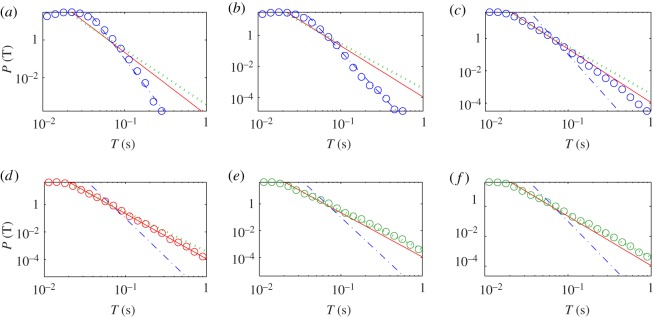
Statistical and scaling behaviours of HFOs, more examples. Distributions of on-interval *T* of IMF 5 (channel 6 of rat 9): (*a*–*f*) for files 1–19, 32–57, 63–72, 78–97, 98–118 and 119–149, corresponding to the pre-stimulation state, post-stimulation state, evolving towards seizure, status epilepticus phase, epilepsy latent period and spontaneous/recurrent seizure period, respectively. The numbers of on-intervals are 354 499, 561 669, 300 291, 458 649, 293 118 and 438 919 for (*a*–*f*), respectively. An algebraic distribution is observed with different exponents for different segments, where the exponents are −5.7, −3.3, −2.9 for dash-dotted line, solid line and dotted line, respectively. The criterion for choosing the threshold $A_{c}$ is the same as in figure 9.

We find that the algebraic scaling law for the on-intervals of HFOs holds for all the animal models. Figure 10*a*–*f* shows more examples for a different rat. In particular, the scaling was calculated for a specific channel for the pre-stimulation state, post-stimulation state, evolution towards seizure, the status epilepticus phase, the epilepsy latent period and the spontaneous/recurrent seizure period, for panels (*a*–*f*), respectively. We see that, while the details of the scaling behaviours can be different for the distinct critical phases (e.g. during epileptogenesis the on-intervals with longer durations are dominant after the first seizure), the algebraic nature of the scaling law is robust.

## 3. Discussion

Seizure prediction, early recognition and blockage of seizures are considered by the membership of the American Epilepsy Society (AES) as the first research priority listed among 15. To achieve these goals a good understanding of the origin, mechanism and dynamics of seizures is necessary. At present the only accessible avenue to probe the origin of epileptic seizures is multiple-channel EEG or ECoG data. Continuous improvement in the experimental methodology has made such data highly reliable and generally of high quality. A challenge is that the amount of EEG or ECoG data is *massive*. An issue of paramount importance and significant interest is to *extract knowledge about epilepsy from data*.

We have developed a method to detect, characterize and analyse the dynamical behaviour of HFOs from a massive database of extensive EEG recordings of a number epileptic rats over two months. We first devise a general and efficient procedure for preprocessing the massive database to exclude erroneous data files and statistical anomalies. (The procedure should also be applicable to massive datasets of other sorts, such as large-scale sensor data or seismic data collections.) We then articulate a procedure based on separating the time scales, the EMD method [23, 24], to detect HFOs. We finally perform a statistical analysis and find evidence for a striking phenomenon: the occurrences of HFOs appear in an on–off intermittent manner and the time intervals that they last exhibit an algebraic scaling.

The methods and results in this paper can potentially be extended to other fields. Big datasets arise not only in biomedicine but also in other fields of science and engineering. For example, in civil engineering, large sensor arrays are often employed to monitor the temperature, humidity and energy flows in large, complex infrastructure systems in a continuous-time fashion. In such an application, the underlying system is in general non-stationary and nonlinear, and to detect behaviours that deviate from the norm or the expected is of considerable interest. Another example is earthquake data. A previous work [58] indicated that seizures can be regarded as ‘quakes of the brain’. It is then conceivable that the idea, method and algorithms developed in this paper can be extended to big seismic database for detecting anomalous oscillating signals (similar to HFOs) preceding the actual occurrence of earthquakes. In general, the EMD/Hilbert transform-based methodology demonstrated in this paper has broad applications because it is specifically designed [23] to deal with nonlinear and non-stationary systems. Owing to its built-in ability to obtain from a complex, seemingly random time series a number of dominant components of distinct time scales, the method is anticipated to be especially effective for anomaly detection. A challenge is to develop mathematically justified, computationally reasonable and automated procedures to detect anomalies, from big datasets, which has been addressed in this paper through the detection of HFOs from massive EEG data with multiple animal models.

We now discuss a number of issues that may warrant future efforts.

First, there is room to improve the EMD-based algorithms developed in this paper, leading possibly to a fully automated method for detecting HFOs and population spikes from massive data for all distinct epileptic stages including pre-stimulation, pre-seizure and recurrent seizures. This will provide a base for probing into the emergence and evolution of HFOs through detailed analyses using methods from nonlinear dynamics, statistics and statistical physics. Special features associated with different types of brain activities can be identified, with the grand goal to exploit the predictive power of HFOs for epileptic seizures. Many questions, which are previously unimaginable, can be asked. For example, does a general class of HFOs exist, regardless of the specific brain activities? What types of HFOs are especially related to seizures? How localized are they around the regions of seizure onset? Are there systematic and characteristic changes in the HFOs during the ictal phase? What types of HFOs are associated with recurrent seizures and is there any relation to the latency period? Answers to those questions and many more will provide a comprehensive picture for the dynamical role of HFOs in epileptogenesis.

Second, with our optimized EMD-based method, HFOs and population spikes can be detected reliably for all the channels. The issue of spatiotemporal evolution of these dynamical events in the brain can then be addressed. For example, we have observed that certain population spikes appear only in some channels at a given time, i.e. they may be highly localized in space. Some HFOs can, however, occur in many channels simultaneously. A possible reason for the dispersive character of the HFOs is that the distance between neighbouring recording sites is about 0.25 mm, which can be within the propagating range of the underlying neuronal activities that cause the pathologic HFOs. The available database from a dense electrode grid thus provides a useful probe to study the propagation of neuronal synchronous activities. For example, by examining the exact timings of HFOs and population spikes and the occurrence of the epileptic seizure in different EEG channels, the propagating pattern of these dynamical events can be determined and, consequently, the sources triggering these events may be identified. A mapping between the epileptic dynamics and activities in different brain regions can be made and the temporal evolution of the mapping can be studied. It would also be useful to examine the correlation patterns of the distinct dynamical events and compare them with those of the background neuronal activities. All these have the potential to provide deeper insights into the origin of epileptogenesis.

Third, in this paper we focused on stable states of the brain, which are the states that last for relatively long periods of time. The transient behaviours were neglected. It would be interesting to study the nonlinear and complex transient dynamics [59] associated with epileptogenesis and brain behaviours in general.

Fourth, there were previous studies of exploiting nonlinear dynamics for analysing epileptic seizures (e.g. [60–66]), and on–off intermittency is an ubiquitous phenomenon in nonlinear dynamical systems [67–82]. It is known in nonlinear dynamics that on–off intermittency can be controlled by applying small but deliberately chosen perturbations to the system [83]. The power-law statistics of the on-intervals associated with HFOs indicate the emergence of a hierarchical organization in the neuronal activities. However, it is difficult to determine uniquely the underlying dynamical mechanism that is distinct from the well-documented mechanism for on–off intermittency. Nonetheless, there are common features between the intermittent behaviours of HFOs uncovered in this paper and those of on–off intermittency, such as scaling properties. If a specific type of HFO can be correlated with the occurrence of seizures and if the underlying dynamics bear similarity to those of generic on–off intermittency, it may be possible to investigate controlling HFO dynamics based on previous works on controlling on–off intermittency. In particular, can the on–off intermittent dynamics of the epileptic HFOs be controlled by using, say, small and infrequent brain stimuli to delay or even eliminate seizures? The results in this paper provide a base for developing computational and experimental schemes to test this control idea.

## 4. Material and methods

### 4.1. Setting of experimental data collection

Experiments were performed on five, two-month old, male Sprague Dawley rats, each weighing between 210 and 265 g. The rats were induced into the state of complete anaesthesia by subcutaneous injection of $10\;\mathrm{mg}\;{\mathrm{kg}}^{-1}$ (0.1 ml by volume) of xylazine and maintained in the anaesthetized state using isoflorane (1.5) administered through inhalation by a precision vaporizer. For each rat, the top of its head was shaved and chemically sterilized with iodine and alcohol. The skull was exposed by a mid-sagittal incision. In three of the five rats, a bipolar, twisted, Teflon-coated, stainless steel electrode (330 μm) was implanted in the right posterior ventral hippocampus (5.3 mm caudal to bregma, 4.9 mm right of midline suture and at a depth of 5 mm from the dura) for stimulating the rat into status epilepticus. The remaining two rats are for control. In all the five rats, a 16 microwire (50 μm, TDT Technologies, Alachua, FL, USA) electrode arraying two rows separated by 500 μm with electrode spacing of 250 μm, was implanted to the left of midline suture horizontally in the CA1–CA2 and dentate gyrus of the hippocampus. The furthest left microwire was 4.4 mm caudal to bregma, 4.6 mm left of midline suture and at a depth of 3.1 mm from the dura. A second microwire array of 16 electrodes was implanted to the right of midline suture in a diagonal fashion. The furthest right microwire was 3.2 mm caudal to bregma, 2.2 mm to the right of midline suture. The closest right microwire was 5.2 mm caudal to bregma, 1.7 mm to the right of midline suture and at a depth of 3.1 mm from the dura. Finally, four 0.8 mm stainless steel screws were placed in the skull to anchor the microwire electrode array: two screws were AP 2 mm to the bregma and bilaterally 2 mm and served as the ground electrodes while two screws were AP −2 mm to the lambdoidal suture and bilaterally 2 mm and served as the reference electrodes. The entire surgical area was then closed and secured with cranioplast cement. Following surgery the rats were allowed to recover for a week.

### 4.2. Data acquisition and file structures

Electrophysiological recordings were conducted by hooking each rat onto a 32-channel commutator, the output of which was fed into the recording system comprising two 16-channel pre-amps, which digitized the incoming signal with a 16 bit A/D converter at a sampling rate of 12 kHz (approx. 12 207 Hz). The digitized signal was then sent over a fibre optic cable to a Pentusa RX-5 data acquisition board (Tucker Davis Technologies). The digital stream of data was stored for later processing. For each channel, the data were recorded and saved as 16-bit signed integer binary files, each of size 600–700 MB (approx. 7.5 h recording time). Thus, for each rat, there were 32 channels, each of which has between 153 and 317 files depending on the recording durations. Each binary file was assigned a *rat* number, a *channel* number and a *file* number. The data were recorded over two months for most of the rats, including pre-stimulation stage, pre-seizure stage, status epilepticus phase, epilepsy latent period and spontaneous/recurrent seizure period.

The sampling rate of the data was relatively high, making it possible to analyse high-frequency, short-duration dynamical events in the brain such as HFOs and population spikes. The typical duration of an HFO is about 100 ms and its characteristic spontaneous frequency can be as high as several hundred hertz. In such a case, our data will have 40 points for a single oscillation period, which is generally enough for various analyses of HFOs. The extensive database provides us with a platform to compare the high-frequency events in different stages in the evolution of epileptic seizures and to systematically investigate the dynamics of epileptogenesis.

### 4.3. Empirical mode decomposition of electroencephalogram data

EMD is specifically designed to deal with nonlinear and non-stationary datasets. In particular, EMD decomposes the signal into a series of IMFs, as follows. For a given signal, EMD determines the local maxima and local minima, and connects them with cubic splines to form an IMF. One then subtracts the IMF from the original signal, and repeats the process to get the second IMF and so on. The procedure is repeated until the remaining signal becomes monotonic. The IMFs are orthogonal to each other (at least locally) and their sum restores the original data. Thus, effectively, the original signal has been decomposed into the IMFs, each in a distinct frequency range, whose non-stationary amplitude and frequency information is well preserved.

Ideally, for given EEG data, the EMD method returns a set of IMFs in separate frequency ranges. Practically, as each data file is too large to be processed computationally, we need to divide the data into small segments so that each segment can be computed within the memory of our computers. To deal with the boundary effect properly, for each data segment, we include an extra but much smaller subset of data points on both ends of the segment, which are from neighbouring segments. These are the corresponding boundary sets. After performing the EMD calculations, only the IMFs within the original data segment are kept, while those associated with the boundary sets are discarded. For a given data segment, the resulting IMFs usually depend on the choices of the sizes of the segment and the boundary sets. In particular, the larger the boundary sets, the more accurate the IMFs, but the amount of the computation will also increase. A systematic test on varying sizes of the boundary sets indicates that the choice of 0.5 s duration (corresponding to 6103 data points at the recording sampling frequency) for each boundary set yields accurate IMFs with tolerable extra computation load. The limited computational power also stipulates that the size of each segment itself cannot be too large. Our systematic test gave 5 s (approx. 61 035 data points) as the optimal duration for balancing the computation time and the reliability of the results. We use the code developed by Rilling [84] to perform the EMD calculations by modifying the original C-Matlab interface to C-codes.

**Figure 11.**
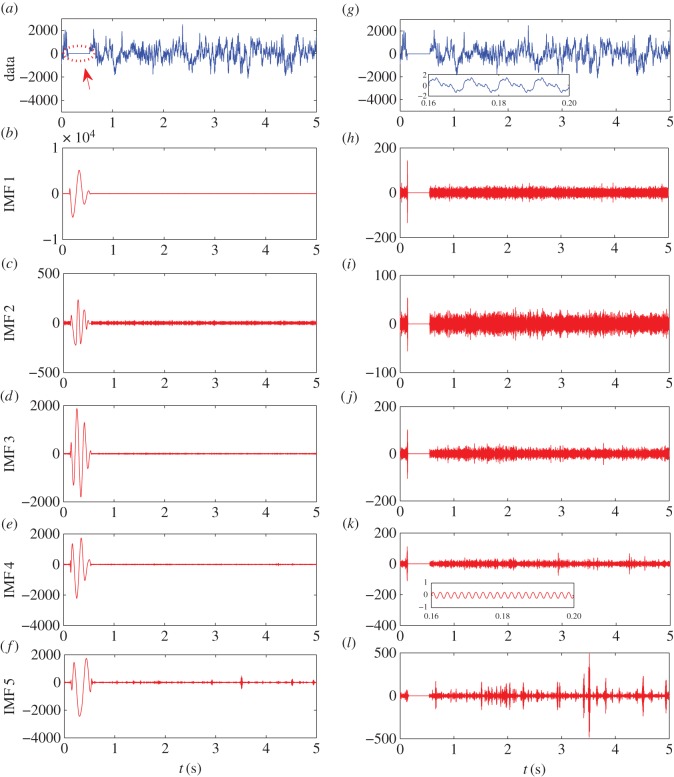
A demonstration of adding small oscillations in EMD computation to eliminate spurious large values in IMFs. (*a*) A 5 s segment of data with about 0.4 s zeros, as indicated by the dotted circle. (*b*–*f*) The first 5 IMFs directly calculated from the data in (*a*). (*g*) Data (*a*) with added small oscillations on the scale of unity (see text), which is almost invisible from the figure. (*h*–*l*) The first 5 IMFs calculated from the data in (*g*). Insets of (*g*) and (*k*) show magnification of the zero region. Note that the scale of the *y* axis is much larger in (*b*–*f*) than those in (*h*–*l*). The anomalies appeared in (*b*–*f*) are effectively removed by the simple method of adding small oscillations to the data segment.

Another practical issue is that the data may contain some discontinuities. In such a case, the EMD program may diverge or have abnormally large values (figure 11*a*–*f*). A remedy is to add a small perturbation in the original signal prior to the EMD calculations. However, due to the difference in the frequency ranges in which the various IMFs lie, small time-varying perturbation signals of frequencies in these distinct ranges are needed. For each frequency range, the amplitude of the perturbation needs to be orders of magnitude smaller than that of the corresponding IMF. For example, if for a normal EEG data segment (denoted by y[i]), there are six IMFs and their frequencies are about 5 kHz, 2 kHz, 1 kHz, 500 Hz, 200 Hz and 100 Hz, respectively (for IMFs 1–6), we will need to add the following small sinusoidal signals: *y*[*i*] = *y*[*i*] + 0.9 × sin (2*πi*/(12 207/100)) + 0.5 × sin (2*πi*/(12 207/200)) + 0.25× sin (2*πi*/(12 207/500))+0.125× sin (2*πi*/(12 207/1000))+0.0625× sin(2*πi*/(12 207/2000))+0.03× sin(2*πi*/(12 207/5000)), where the amplitudes of the IMFs are typically larger than 10. The perturbation signals thus will not have any practical influences on the IMF results for normal data. However, when there is a discontinuity with a linear relaxation in time, the corresponding IMFs will contain the added small sinusoidal oscillations instead of generating divergence or large anomalies (figure 11*g*–*l*). In addition, when the original data is contaminated by a small segment of zeros, without adding the small oscillations, the resulting IMFs will oscillate wildly in this region with amplitudes orders of magnitude larger than those of the normal datasets (figure 11*a*–*f*). This is because, for obtaining each IMF, EMD looks for the local maxima and local minima and then approximates the data with cubic spline connecting the maxima or minima. When a segment of zeros is encountered, there are no local maxima or minima so that the EMD extrapolates with cubic spline using the maxima or minima outside this region. For the first IMF, as the frequency is the highest (approx. 5 kHz), even a zero segment of about 0.1 s would correspond to about 500 maxima or minima. Thus, the extrapolation will generate extremely large, artificial oscillations. The remainder obtained by subtracting IMF 1 from the original data will compensate the large oscillations in IMF 1, but they will propagate to subsequent IMFs. The conclusion is that, adding the small sinusoidal perturbing signals causes essentially no difference in the original signal (about 1 part in 1000), but the artificial anomalies can be effectively eliminated.

## Data accessibility

All data used in the study have been uploaded onto Google Drive and are publicly available: https://drive.google.com/drive/folders/0B7S5nQOU_nMIYkEyYmJvTGpNOVU?usp=sharing.
